# On Cases of Encephalitis Lethargica in Bristol

**Published:** 1920-03

**Authors:** D. S. Davies, J. O. Symes, F. H. Edgeworth, I. Walker Hall, J. A. Nixon


### ON CASES OF ENCEPHALITIS LETHARGICA IN BRISTOL.

BY

D. S. Davies, J. O. Symes, F. H. Edgeworth,
I. Walker Hall and J. A. Nixon.

In this paper an attempt is made to survey the general characteristics of the recently identified disease encephalitis lethargica, as manifested in the Bristol outbreak of 1919.

The paper is a composite one, and the various points of view of the contributors serve to illustrate and define the salient features of the disease. Dr. D. S. Davies describes the disease in relation to questions of public health; Dr. Symes records a case in which, apparently, direct infection took place; Dr. Edgeworth contributes an analysis of the clinical features of the twenty cases reported, and following this is an account, by him and Dr. Walker Hall, of three cases which throw light on certain clinical and pathological aspects of the disease. Lastly, Dr. Nixon reports on a case occurring as far back as 1908, which, obscure at the time, can now be identified as one of lethargic encephalitis.

In the spring of 1918 the occurrence of an obscure disease, associated with progressive languor and muscular weakness, passing into lethargy and disablement, and with various cranial nerve affections, especially ptosis, strabismus and nystagmus, was noted in several centres of population, and a limited number of cases were reported in Bristol under the assumption that they were cases of "botulism," a rare food-poisoning condition originally described by Van Ermengem. There is little doubt, in the light of recent knowledge, that these were cases of encephalitis lethargica, and the year 1919 furnished further evidence of its existence.

During this year twenty cases were reported under this heading, and though the number is far too small to render any statistical conclusions accurate, it may be useful to consider them in detail, and to ascertain how far they agree with results observed elsewhere.

*Sex Distribution.*—Of the twenty cases eleven were males and nine females. This nearly resembles the ratio of males to females (3–2) observed in epidemics of *poliomyelitis;* the figures recorded for *encephalitis lethargica* are in the ratio of 68 males to 58 females.

*Age Incidence.*—

| | M. | F. |
|---|---|---|
| 0–10 | 3, 8, 9. | 3. |
| 10–25 | 14, 18, 21. | 16, 16, 16, 16, 23. |
| 25–35 | 31. | 26, 29. |
| 35–45 | 35, 41. | 37. |
| 45–55 | 48, 52. | |
| | 11. | 9. |
| | Total 20. | |

Various ages from 3 to 52 furnished cases, ages under 35 yielding 76 per cent. and higher ages 24 per cent. of the total.

No cases were notified in infants under 1.

The marked incidence in females aged 16 is notable, and this age group 10–25 yielded no less than 42 per cent. of the cases.

*Occupation and Social Status.*—Nothing causally suggestive arises either from the occupations followed or the social position of the patients.

In the case of the laundry-maid employed at a General Hospital, no recent case had been nursed in the Hospital. This maid lived in Hospital, but her home address was in South Bristol, in a district from which no cases were reported.

*Seasonal Incidence.*—Colonel S. P. James found that most of the cases (126) he reported in England occurred during the seven weeks between March 16th and May 4th, the disease reaching its maximum prevalence somewhat slowly, maintaining this prevalence for a comparatively brief period, and gradually declining.

The Bristol cases showed the following distribution, cases occurring regularly from December 18th, 1918, to May 19th, 1919, then after a four months' interval recommencing in October :—

| 1918. Dec. | 1919. Jan. | Feb. | Mar. | April. | May. | June. |
|---|---|---|---|---|---|---|
| 3 | 5 | 4 | 2 | 1 | 1 | — |

| July. | Aug. | Sept. | Oct. | Nov. | Dec. |
|---|---|---|---|---|---|
| — | — | — | 1 | 1 | 2 |

There is evidence only in one instance of limited power of infectivity, viz. the daughter (Case 10) who sickened sequentially to her father (Case 9). This is reported below by Dr. Symes. In all the other instances reported the cases are " sporadic," and bear no obvious relation to each other as regards occupation or association.

It appears, indeed, that encephalitis lethargica belongs to that group of maladies (including also cerebro-spinal fever and acute poliomyelitis) in which the pathological agent is more frequently present in the human organism than the clinical symptoms indicate. A manifestation of the disease occurs either as the result of a breakdown of the immunity to the effects of the virus, which the individual who harboured it had up to that time enjoyed, or as the result of a non-immune person becoming infected with a strain of the virus which has attained the degree of pathogenicity described as specific. In inter-epidemic periods both the conditions named are in favour of the individual, thus clinical cases become very occasional or sporadic. The cases here reported were for the most part thinly scattered over various districts, working class or well-to-do.

The group of four (4, 14, 15, 16) occurred at irregular intervals from January to May, 1919, and afford no evidence of inter-communication. The group of 1 and 11 were also separated by an interval of a month. Twelve cases were removed to hospital: six cases to the Royal Infirmary, five to the General Hospital, one to Novers Hill Hospital. Of the nine fatal cases four died in hospital. The age and sex of the fatal cases are :—

| *Males.* | *Death after Onset.* | *Females.* | *Death after Onset.* |
|---|---|---|---|
| 3 years. | 19 days. | $3\frac{3}{4}$ years. | 25 days. |
| 8 ,, | 14 ,, | 16 ,, | 10 ,, |
| 9 ,, | 14 ,, | 23 ,, | 22 ,, |
| 14 ,, | 3 ,, | 26 ,, | 3 ,, |
| 18 ,, | 12 weeks. | | |

D. S. D.

Multiple cases of encephalitis lethargica in the same house have been recorded but are not common, and a history of infection from direct contact with a sufferer from this disease is very seldom obtained. MacNalty reports the case of a pregnant woman who developed encephalitis. Labour was induced and a healthy child was born. "A few days later" the infant developed the disease. Both mother and child recovered. The same writer reports three cases in one family at Mansfield. The first patient, a female child aged $1\frac{3}{4}$ years, became ill with encephalitis on April 19th, her brother, aged nine, was attacked on May 4th, and a second brother, aged 7, on May 8th. Two of the children died. He also records two other cases, in one of which the incubation period was probably fourteen days and in the other sixteen days.

Reece reports a fatal case in a boy who was attacked on

May 19th, and died May 21st. His sister was attacked May 23rd, and died May 24th. Other cases quoted by Reece are: Man attacked December 7th, died January 19th—he was nursed by his wife, who sickened January 14th and died January 19th; a lady taken ill on December 5th was removed to a nursing home December 17th—her night nurse at the home developed encephalitis about December 26th or 27th.

The cases of encephalitis lethargica under my care which seem to throw some light on the subject of the incubation period are Nos. 9 and 10 of the series now reported. The father, who lived a few miles out of Bristol, after an indefinite illness at home, was removed to the General Hospital on February 5th, 1919, and found to be suffering from encephalitis lethargica. His daughter worked and lodged in Bristol, but visited her home and saw her father on the day of his removal to hospital. She complained of headache and tiredness on February 8th and 9th, but returned to her work on the 10th and 11th. The febrile attack started on February 12th, she was removed to the hospital on February 17th and died on the 20th. This would give an incubation period of seven days.

From a consideration of the foregoing it seems evident that, although the infectivity of encephalitis is a very low one, yet the disease is capable of transference from person to person; that it has a short incubation period of from four to seven days, but that the specific organism may lie latent for a much longer period in persons who are contacts.

J. O. S.

An analysis of the clinical features of the cases reported shows that in five cases the disease had been preceded by influenza, at intervals varying from some weeks to a few months. In view of the prevalence of influenza in Bristol during the autumn and winter of 1918–19, these numbers do not suggest any causal connection between the two diseases.

Malaise and headache, generally unilateral, were the most common initial symptoms. In three cases vomiting occurred. Fever came on and lasted from a few days to three weeks, the patient becoming more and more lethargic, and often delirious at night. A rash appeared in three cases—urticarial, or macular, or papular. In only one case did the nervous symptoms consist solely of lethargy. In the other nineteen cases some affection of one or more cranial nerves appeared. In three cases nystagmus only was present, whilst in six cases there was some ocular paralysis, either unilateral or bilateral, varying from paresis of one muscle to total ophthalmoplegia. In eight cases some ocular muscle affection was accompanied by paresis or paralysis of the facial muscles, generally unilateral in distribution, more rarely bilateral. In one case a facial paresis, unaccompanied by any ocular affection, occurred. In two cases paralysis of the soft palate, associated with ocular and facial paresis, occurred. Aphasia was noted in three cases. In four cases the tendon jerks were lost, without any definite palsy. In one case paresis of the right arm and right leg occurred, with loss of the tendon jerks; this was the only case in which any definite polyneuritic phenomena appeared. Tremor, unilateral or bilateral, was present in three cases, and in one a general convulsion took place during the fever.

On comparison of these phenomena, observed in Bristol, with those described by MacNalty it would appear that all his types of the disease occurred, with one exception—no case of cerebellar ataxy was noted.

Nine of the twenty cases notified in Bristol died. This gives a mortality of 45 per cent. MacNalty found a mortality of 22 per cent. in 139 cases. The discrepancy of the Bristol figures is probably due to the small number of cases; it can hardly be taken as indicating a more serious form of the disease in Bristol than that which occurred in London and the Midlands in 1918.

The diagnosis of the disease may be easy or difficult. It has to be remembered that, in the absence of lethargy, no cranial nerve paralysis can be regarded as evidence of its presence. Further, optic neuritis, retraction of the head and Kernig's phenomena must be excluded. If that can be done, as is easily possible, the majority of the pitfalls in diagnosis can be avoided. The most likely source of error is tuberculous meningitis.

F. H. E.

Frank M., aged 3, was admitted to the Royal Infirmary on January 4th, 1919, and died on January 12th, 1919. He had been brought to Bristol from London three months previously, had fallen ill on December 24th with headache and vomiting and subsequently became irritable. On December 30th he became drowsy, and then partially unconscious. On admission on January 3rd he was semi-comatose, with dilated pupils and incontinence of urine. The abdomen was scaphoid in shape, and the legs drawn up. Some erythematous maculæ were visible on the arms. He rolled his eyes about, but the right one less than the left. Kernig's sign and head-retraction were absent. The optic discs were normal. The cutaneous reflexes were normal, the knee-jerks absent. Spinal puncture showed a clear cerebro-spinal fluid, not under pressure. It contained a few cells, which on a differential count showed polymorphs 10 per cent., mononuclears 70 per cent., endothelials 20 per cent. It was sterile. On January 8th examination of the blood showed 10,320 white corpuscles per cb.mm. On January 9th left ophthalmoplegia developed, and on the following day left facial paralysis and nystagmus of the right eye. The temperature remained normal until the morning of January 12th, when it rose to 103.6 just before death.

The only *post-mortem* changes visible to the naked eye were congestion of the lower lobes of both lungs. On microscopical examination the cortex of the anterior and posterior lobes of the cerebrum showed general œdema and a few hemorrhages. There was a perivascular infiltration of a few blood-vessels by mononuclear cells. Here and there a capillary was thrombosed. The nerve-cells were unaffected, and no proliferation of the neuroglia was found. In the floor of the 3rd and 4th ventricles, pons, medulla and spinal cord, the hemorrhages were more numerous and arranged superficially; the blood-vessels were more generally infiltrated by mononuclear cells, and around some there were definite foci of these cells. The tissue immediately underlying the floor exhibited a layer of mononuclear cells, and the perivascular tissues were thickly infiltrated. In the anterior horns of the cervical portion of the spinal cord were definite foci of mononuclear cells. The nuclei of occasional nerve cells showed shrinking and eccentric positions. Chromatolysis was not a general feature. No foci of proliferation of the neuroglia were seen. In the heart slight cloudy swelling and evidence of irritation of the perivascular tissues were visible. The lungs showed catarrh of the small bronchi and general œdema, the kidneys cloudy swelling, and the liver general fatty infiltration.

Ellen D., aged 16, was admitted on January 17th, 1919, and died January 21st, 1919. Her mother said that the girl had fallen ill one week previously with headache, and had subsequently become delirious. On admission the temperature was 102°, pulse 126, and respiration 24 per minute. She was lethargic and did not move spontaneously, except that she talked incoherently. Occasionally she answered questions, saying that she had pain in her neck and abdomen. The only abnormal physical sign, other than those recorded above, was lateral nystagmus of both eyes on looking to the right or left, which she did on request. Spinal puncture showed a clear cerebro-spinal fluid, not under pressure, containing hardly any cells, and sterile on culture. Examination of the blood showed 25,000 white corpuscles per cb.mm. On a differential count polymorphs 65 per cent., large lymphocytes 12 per cent., small lymphocytes 17 per cent., hyalines 3 per cent., and eosinophils 3 per cent. were found. On January 19th she was more comatose, and lay like a log in the bed. The temperature remained high until death on January 21st.

A *post-mortem* examination showed a marked turgescence of the superficial cortical veins, with some hemorrhages in the pia-arachnoid. Microscopical examination showed a few scattered minute hemorrhages, with perivascular infiltration of mononuclear cells in the cortex of anterior and posterior cerebral lobes. The nerve cells were unaffected, and no proliferation of the neuroglia was found. In the floor of the 3rd and 4th ventricles, pons, medulla and spinal cord there were numerous perivascular cell-infiltrations with occasional hemorrhages. Superficially there was a definite layer of mononuclear cells and irritated blood-vessels. The anterior horns of the cervical cord showed many mononuclear perivascular foci.

There was some diminution of nuclei and alteration of their position in the nerve cells. No proliferative foci of neuroglia were seen. In the heart there was evidence of considerable perivascular irritation, and cloudy swelling of the muscle cells. Some bronchiolitis and œdema were found in the lungs, fatty infiltration in the liver, and early catarrhal nephritis in the kidneys.

May R., aged 16, was admitted on January 21st, 1919, and went out well on March 17th, 1919. She had previously, in August, 1917, been a patient (of one of us) at the Infirmary with a rapidly-developed complete paraplegia, with no prominence of any vertebral spine, but with X-ray evidence of tuberculous disease of the lower dorsal vertebræ. With rest in bed and a plaster jacket she had recovered completely. About January 12th she complained of pain in the neck, and a little later became delirious, with incontinence of urine and fæces. On admission the temperature was 97°, pulse 96, and respiration 24 per minute. She lay motionless in bed, with mask-like face and closed eyes. She answered questions in an indistinct, slurred voice. There was no head retraction nor Kernig's sign. Both plantar reflexes were extensor in type, the left knee-jerk was active, while on the right side rectus and ankle-clonus could be elicited. [These abnormal signs in the legs were probably the remains of the paraplegia—evidence of some permanent structural damage to the pyramidal tracts, though the function had been completely restored.] She had opened her eyes on request. The pupils were equal and contracted. The optic discs were normal. Nystagmus was present in both eyes on lateral or vertical movement. Lumbar puncture showed a clear cerebro-spinal fluid, not under pressure; on centrifugalisation it showed a few cells, too few to count. 35,000 white corpuscles per c.mm. were found in the blood, and, on a differential count, polymorphs 70 per cent., small lymphocytes 17 per cent., large lymphocytes 9 per cent., hyalines 1 per cent. and eosinophils 3 per cent. were found. The patient became more lethargic, and a fortnight later no response was obtained on speaking to her. From that time onward she slowly improved. On February 23rd the nystagmus had disappeared, and she began to take notice of people in the ward. The number of leucocytes was 20,000 per c.mm. On March 13th they numbered 14,300. On March 17th she went home quite well.

MacNalty, in his most excellent Report to the Local Government Board on Lethargic Encephalitis, stated that it is "a general infectious disease characterised by manifestations originating in the central nervous system, of which the most frequent and characteristic are progressive lethargy, and a lesion in or about the third pair of cranial nerves."

The three Bristol cases are of some interest in regard to the evidence they gave in support of this opinion, both in the widespread distribution of the lesions and in the occurrence of leucocytosis. From the increasing records of other cases and outbreaks it is becoming apparent that there is a wider distribution of the ætiological factor in the tissues. Marinesco mentions arachnoidal changes also, and Fairbourn describes the occurrence of a few cortical hemorrhages in his case. Tucker observed dilated cortical capillaries with slight aggregations of polynuclears in the perivascular spaces. Wegeforth and Ayer found occasional blood cells and granule - laden phagocytes in the spaces immediately surrounding the vessels in the cortex. The same authors state that there was some perivascular exudate in the cervical cord, a condition we noted in both our cases. Marinesco noted a patchy meningo-myelitis of the cervical cord in his cases. These findings are all in accord with MacNalty's type F of the disease, in which he considers there is evidence of spinal involvement. Confirmation of these facts is available from the experimental work of Lœwe, Hirschfield and Strauss. These workers obtained definite cortical changes in many of their experimental animals.

With regard to the other organs, Wegeforth and Ayer examined the heart, intestine and lungs in one case, but failed to find any perivascular changes. They state, however, that the affected vessels showed intimal proliferation. In both our cases the cardiac vessels exhibited perivascular changes, and the liver, lungs and kidneys presented morbid lesions.

It is perhaps worthy of recall that two of our cases showed definite leucocytosis in the fourth and eighth day respectively, together with a slight relative increase in the lymphocytes. In one instance there was pyrexia, in the other the temperature was normal. This early leucocytosis is in accord with the findings in the American cases. It is now recognised that the white cells tend to decrease after the first week of the attack.

F. H. E., I. W. H.

In 1908 a boy came under my care at the Bristol Royal Infirmary whose case aroused much interest at the time. The patient was shown at the Bristol Medico-Chirurgical Society, and the diagnosis then agreed upon was "Polio-encephalitis, with possible involvement of the cerebello-rubro-spinal tracts." No similar cases had been observed in the district.

The patient was a healthy, well-developed boy of seven, living in Bristol. He had had no previous illness except a mild attack of measles some years before. His parents were alive and well, with four other children all healthy. His illness began on September 28th, 1908, with vague malaise. On October 2nd he was admitted to the Bristol Royal Infirmary complaining of pain in the head, which he located over the left eye. It was said that he had been drowsy all the previous week. On the day before his admission he began to speak with a stutter. His left arm and his left leg seemed to become rigid. He had no fits or vomiting.

For a fortnight or more the boy lay in a lethargic condition, taking no notice of anyone. He answered no questions, but seemed to understand everything said to him. At first he took food with difficulty, but later nasal feeding was necessary as the teeth were kept tightly clenched. There was no head retraction, Kernig's sign was absent, knee-jerks present and fairly active, a Babinski reflex on the left side.

The pupils were unequal, the right being dilated. Nystagmus was present, but no optic neuritis. There was some loss of power in the left arm. The cerebro-spinal fluid was clear, with hardly a cell to be seen, and no organisms were found in smears or cultures. Lumbar puncture was performed three times. There was a moderate degree of pyrexia (100°–101° F.), the temperature fell by lysis and remained normal after October 16th.

By October 20th the boy's condition had become much worse. He lay perfectly still all day unable to take or swallow his feeds. He was rigid in all his limbs, with marked head retraction. On October 30th there was great improvement. He was able to understand when spoken to, had spoken and smiled. The rigidity was less marked.

After this time he slowly recovered, but it was noticed that he held his hands and fingers in a position suggesting the carpal spasm of tetany. This was not present in the feet. He also developed a constant tremor in the head and hands, much increased on voluntary movement. He gradually grew stronger. His mental and intellectual capacity appeared completely restored. Eventually the boy left the Infirmary in the condition of a case of well-marked "paralysis agitans," with shaking head, "cigarette-rolling" fingers, a forward stoop and rigid shuffling gait. There was excessive rigidity in all muscles. I have endeavoured to trace the boy and find out his present condition without success.

There seems no reason to doubt that this was a sporadic case of encephalitis lethargica. The characteristic triad of symptoms was present: fever, lethargy and lesion of cranial nerve nuclei or tracts in the brain-stem. The absence of optic neuritis and of Kernig's sign, together with the negative findings in the cerebro-spinal fluid, agree with our later observations on this disease. Recent observers have recorded other instances in which a condition resembling paralysis agitans has occurred in non-fatal cases of encephalitis lethargica. I have at the present time a girl, aged 8, in the Royal Infirmary in whom the disease has run an almost identical course.

